# Regulation of Axillary Meristem Initiation by Transcription Factors and Plant Hormones

**DOI:** 10.3389/fpls.2016.00183

**Published:** 2016-02-18

**Authors:** Minglei Yang, Yuling Jiao

**Affiliations:** State Key Laboratory of Plant Genomics, Institute of Genetics and Developmental Biology, Chinese Academy of Sciences, and National Center for Plant Gene ResearchBeijing, China

**Keywords:** axillary meristem, auxin, cytokinin, transcription factors, post-embryonic development

## Abstract

One distinctive feature of plant post-embryonic development is that plants can undergo reiterative growth and continuous organogenesis throughout their lifetimes. Axillary meristems (AMs) in leaf axils play a central role in this growth and differences in meristem initiation and development produce the diversity of plant architecture. Studies in the past 15 years have shown that several transcription factors (TFs) and phytohormones affect AM initiation. In this review, we highlight recent research using systems biology approaches to examine the regulatory hierarchies underlying AM initiation and the role of auxins and cytokinins in AM initiation and development. This research revealed a developmental mechanism in which phytohormone signals act with a gene regulatory network containing multiple TFs to contribute to the initiation of AMs.

## Axillary Bud Formation and Lateral Branching

Plant post-embryonic organs originate from meristem tissues, which contain pluripotent cells. During embryonic development, the shoot apical meristem (SAM) and root apical meristem establish the primary axis of the plant and subsequently give rise to shoot and root structures (Pautler et al., 2013). In seed plants, the SAM repeatedly produces phytomers consisting of a leaf, an axillary meristem (AM), and an internode. Each AM functions as a new SAM to establish a secondary growth axis from a lateral bud situated in or near the leaf axils. Thus, SAMs and AMs produce the overall architecture of the plant (Pautler et al., 2013).

Generally, development of axillary buds comprises two stages, initiation in the leaf axil, and subsequent outgrowth or dormancy (Tantikanjana et al., 2001). During vegetative growth in *Arabidopsis thaliana*, axillary buds initiate at a distance from the SAM and establish an acropetal gradient of axillary bud formation (Hempel and Feldman, 1994). During the reproductive phase, axillary buds initiate near the SAM and form in leaf axils of the youngest primordia where they establish a basipetal pattern of initiation and outgrowth (Hempel and Feldman, 1994). Axillary bud formation seems to undergo differential regulation during vegetative and reproductive phases. AM initiation depends on the leaf axil stem cell niche and establishment of this niche is tightly linked to the formation of the boundary region between the stem and the leaf primordium. In addition, AM initiation is also closely associated with leaf polarity because AMs only develop at the adaxial base of the leaf, facing the SAM (McConnell and Barton, 1998).

Most mutants with altered patterns of shoot branching, including *auxin resistant* and *more axillary growth* mutants in *Arabidopsis* (Leyser, 1997; Stirnberg et al., 2002), *decreased apical dominance* mutants in petunia (*Petunia hybrida*) (Napoli et al., 1999), and *ramous* mutants in pea (*Pisum sativum*) (Napoli et al., 1999), exhibit increased axillary bud outgrowth activity. Auxins and strigolactones inhibit axillary bud outgrowth (McSteen and Leyser, 2005; Wang and Li, 2008); by contrast, cytokinins antagonize auxins and strigolactones to promote axillary bud outgrowth (Gomez-Roldan et al., 2008; Janssen et al., 2014). Intriguingly, recent work reported that sugar regulates axillary bud outgrowth, which may explain apical dominance (Mason et al., 2014).

In recent years, a combination of mutant analysis, imaging, and systems biology approaches have been used to understand the process of AM initiation. This review aims to integrate current knowledge about the regulation of AM initiation, especially the role of transcription factors (TFs) and phytohormones.

## Axillary Meristem Initiation in *Arabidopsis*

**FIGURE 1:**
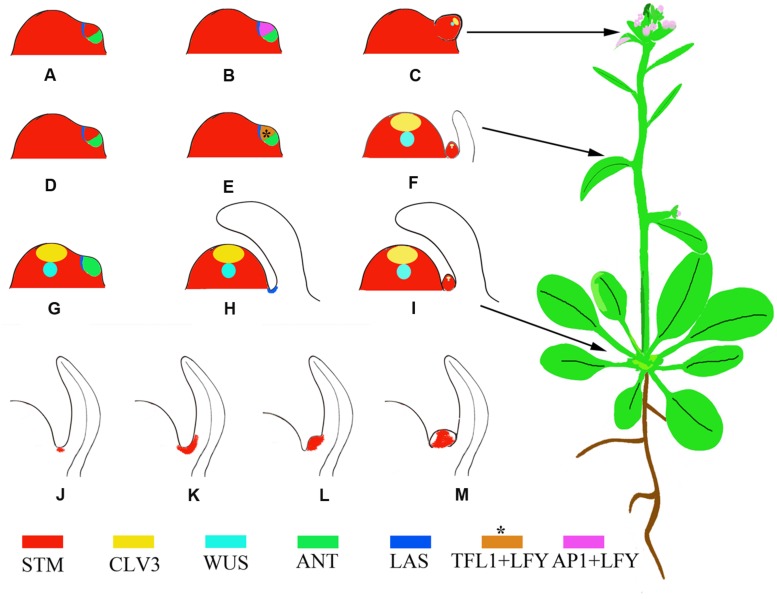
**Schematic representation of phytomers with different developmental fates and AM developmental stages marked by the expression of *STM*.** **(A–C)** A reproductive primordium develops as a bias in the meristem suppresses expression of leaf domain genes. Instead, the meristem maintains *STM* expression and high levels of *LFY* and *AP1* expression (Grbic, 2005). **(D–F)** A transitional primordium develops leaf and axillary meristems (AMs) concomitantly. The floral meristem identity gene *LFY* and the shoot meristem identity gene *TFL1* (brown color) are expressed at high levels in this primordium (Ratcliffe et al., 1999), which confers mixed features of both leaf and flower primordia. This primordium develops into a cauline phytomer. **(G–I)** A vegetative primordium develops with the leaf domain prevailing, due to biases toward leaf fate, resulting in a rosette phytomer. **(J–M)** AM initiation in the leaf axils. The blank region of **(M)** without *STM* expression indicates primordium initiation. Asterisks indicate that the expression pattern is postulated and not verified by experiments.

AMs initiate during the vegetative growth phase; after the floral transition, floral meristems (FMs) initiate to replace leaves. Analysis of molecular markers indicated that the FM is a specialized AM where the leaf degenerates into a bract or cryptic bract after the floral transition (Long and Barton, 2000). Based on this theory, a lateral organ likely consists of two domains corresponding to the emerging leaf and the AM. These two domains are marked by the expression of the leaf markers *AINTEGUMENTA* (*ANT*) and *ASYMMETRIC LEAVES 1* (*AS1*) and the meristem marker *SHOOT MERISTEMLESS* (*STM*), respectively. Rosette leaves, cauline leaves, and FMs, which form during the vegetative, transition to reproductive, and reproductive growth phases, respectively, have different partitions of these two domains (**Figures 1A–I**) (Grbic, 2005). Whereas the mechanism of bract suppression in *Arabidopsis* flower has been well studied (Whipple et al., 2010), how the partitions of domains change accompanying phase transition requires further studies.

During vegetative development, each phytomer consists of a large rosette leaf, a short stem segment (internode), and an initially morphologically undetectable AM (McSteen and Leyser, 2005). AMs initiate at the junction between the stem and the adaxial base of the subtending leaf. Cells in leaf axils are smaller in volume than their neighbors and are presumably meristematic at this stage. The expression of *STM* can be detected in leaf axils of all stages (**Figure 1J**) (Grbic and Bleecker, 2000; Long and Barton, 2000; Greb et al., 2003), although it remains to be tested if a cell population that continuously expresses *STM* exists. Because the *STM*-expressing cells ‘move’ upward toward the leaf, the initial *STM*-expressing cells may create a separation while their neighboring cells re-differentiate as AM progenitor cells (Long and Barton, 2000). The *STM*-expressing meristematic cells decrease in number during leaf growth and finally become restricted to the center of the leaf axil. The first visible change in the leaf axil is the appearance of a group of dense-staining, fast-dividing cells surrounded by the beneath shell zone cells (Shah and Patel, 1972; Grbic and Bleecker, 2000; Wang et al., 2014b), a well-developed, curved zone of elongated, cambiform, and vacuolated cells that delimits the meristematic cells from the adjacent tissue. Prior to axillary bud formation, *STM* expression is up-regulated in meristematic cells (**Figure 1K**) (Long and Barton, 2000; Greb et al., 2003). Subsequent division of the meristematic cells in the leaf axil leads to the morphologically distinguishable bump on the adaxial leaf base (**Figure 1L**) and eventually a visible axillary bud forms having its own leaf primordia (**Figure 1M**).

Following the floral transition of the SAM, the two or three leaf primordia formed just prior to the transition develop into cauline leaves. The cauline leaves are smaller than rosette leaves and are associated with actively growing axillary buds (Hempel and Feldman, 1994). The AMs subtended by cauline leaves develop concomitantly with leaves (Grbic, 2005). Both *TERMINAL FLOWER1* (*TFL1*), which inhibits FM fate, and *LEAFY* (*LFY*), which promotes FM fate, are expressed during this period (Ratcliffe et al., 1998; Grbic, 2005) (**Figures 1D–F**).

After the floral transition, the SAM is termed an inflorescence meristem and forms primordia with floral fates. Each primordium develops into an FM subtended by a cryptic bract and has an elongated internode. The morphologically invisible cryptic bract can be detected as a region that expresses *ANT* but not *STM* and is considered to have similarities to a leaf primordium. In the primordium, *ANT* expression ceases at an early stage, leading to a failure of bract growth (Long and Barton, 2000).

Von Goethe proposed that a flower can be considered a compressed shoot (Dornelas and Dornelas, 2005). However, a flower differs from a shoot in its determinate growth, which results from transient AM activity and produces a limited number of floral organs. In addition, a flower does not branch because its floral organs, like sepals, do not have AMs. *APETALA1* (*AP1*) and homologous MADS-family TF genes mediate the suppression of sepal axil AM formation and mutations of these genes result in secondary flowers in sepal axils (Irish and Sussex, 1990; Mandel et al., 1992; Sundstrom et al., 2006). The regulation of secondary FMs of a sepal axil shares similarity with the regulation of vegetative AMs (Han et al., 2014).

## Transcription Factors in Axillary Meristem Initiation

Most of the known genes that affect AM initiation encode TFs. AMs initiate in the leaf axils, closely associated with the boundary zone that separates the leaf primordium from the shoot meristem. Many of the genes that affect AM initiation also influence boundary formation (Wang et al., 2016), although genes affecting boundary formation do not necessarily affect AM initiation.

*LATERAL SUPPRESSOR* (*LAS*) encodes a member of the GRAS family of TFs (Greb et al., 2003), and *Arabidopsis las* mutants have vegetative stage-specific defects in AM initiation. *LAS* homologs also affect reproductive stage development in tomato (*Solanum lycopersicum*) and rice (*Oryza sativa*) (Groot et al., 1994; Schumacher et al., 1999; Li et al., 2003). *LAS* transcripts specifically accumulate in the axils of primordia, which derive from the SAM during vegetative and reproductive development (Greb et al., 2003).

*REVOLUTA* (*REV*) encodes a class III homeodomain/leucine zipper TF (HD-ZIPIII). Unlike *las* mutants, *rev* mutants show reduced AM formation in both vegetative and reproductive stages (Otsuga et al., 2001). In addition, *REV* has a broad expression pattern in many tissue types in shoot and root. In addition to AM initiation, *rev* mutants show defects in the development of the SAM, leaves, vasculature, and roots.

Members of the redundant family of *REGULATOR OF AXILLARY MERISTEMS* (*RAX*) genes encode Myb-like TFs and affect AM initiation in *Arabidopsis* and in tomato (Keller et al., 2006). Compared to *LAS*, expression of *RAX1* is spatially more restricted at the center of the leaf axil and *RAX1* is the earliest known marker of the position of the AM (Keller et al., 2006).

In addition, NAC domain proteins CUC1, CUC2, and CUC3 have redundant but partially distinct functions in boundary formation and AM initiation. The *CUC* genes have boundary-specific expression, but different functions. *CUC1* shows no obvious role in AM initiation, *CUC2* slightly affects AM initiation, and *CUC3* functions as a major regulator of AM initiation (Hibara et al., 2006). Compared with the single mutants, the *las cuc3* double mutants exhibit more frequent axillary bud defects, suggesting that *LAS* and *CUC3* have overlapping functions in AM initiation.

**FIGURE 2:**
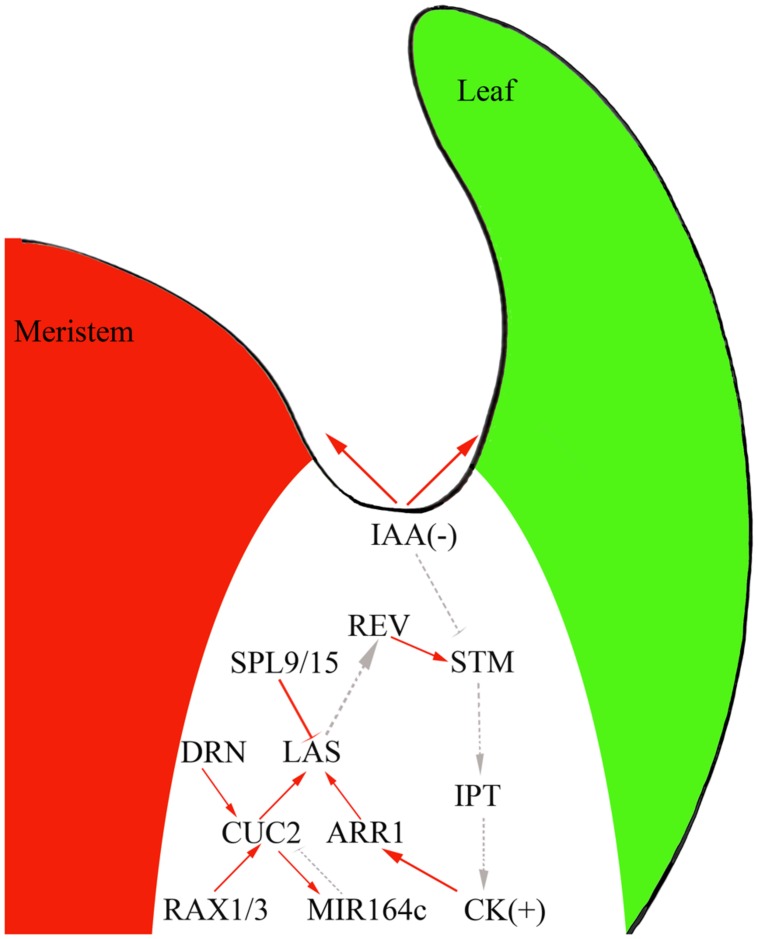
**The interplay of phytohormones and transcription factors (TFs) to control AM initiation in the boundary domain.** Red solid line, direct interaction; gray dotted line, genetic interaction; arrow, activation effect; bar, repressive effect.

Genetic studies and genome-scale analyses have begun to uncover the gene regulatory network (GRN) underlying AM initiation. For example, *CUC* genes regulate *LAS* expression (Hibara et al., 2006; Raman et al., 2008) and CUC proteins directly bind to the *LAS* promoter and activate its expression (Tian et al., 2014). Cell type-specific gene expression analysis and genome-scale yeast-one-hybrid assays showed that *CUC2* and *LAS* function as two hubs of this GRN, with their promoter regions connected to many TFs (Tian et al., 2014). For example, *RAX1* and *3* directly activate *CUC2*; CUC2 then directly activates the expression of the miRNA gene *MIR164c* and miR164 degrades *CUC1* and *2* transcripts to form a regulatory loop (Laufs et al., 2004; Mallory et al., 2004; Larue et al., 2009; Tian et al., 2014). GRN analyses have also identified new regulators of AM initiation. For example, *DORNRÖSCHEN* regulates AM initiation through direct activation of *CUC2* (Tian et al., 2014). SQUAMOSA-PROMOTER BINDING PROTEIN-LIKE proteins suppress AM initiation by directly repressing *LAS* expression (**Figure 2**) and thus connect the aging pathway to AM initiation.

## Phytohormones in Axillary Meristem Initiation

Auxin and cytokinins have broad and vital effects on plant development and recent studies have suggested that these phytohormones also regulation AM initiation. Indeed, experiments using the DII-Venus auxin sensor (Vernoux et al., 2011) showed that the leaf axil region where AMs initiate has low auxin concentrations (Wang et al., 2014a,b). AM initiation requires this auxin minimum; ectopic production of the auxin biosynthesis enzyme iaaM in the leaf axil can lead to compromised AM initiation. In leaf axils, the expression of the meristematic cell marker *STM* decreases, but the expression of *LAS* remains unchanged (Wang et al., 2014b). The leaf axil auxin minimum depends on auxin efflux and influx. In *pin-formed1* mutants, which affect a key auxin intercellular efflux transporter, and *aux1* mutants, which show compromised auxin influx, the auxin minimum in the leaf axil disappears and AM initiation fails to take place (Wang et al., 2014a,b).

Following the formation of the auxin minimum, cytokinin accumulates at the leaf axil (Wang et al., 2014b) as detected by the synthetic reporter p*TCS*:*GFP-ER* (Muller and Sheen, 2008). The leaf axil cytokinin signaling pulse depends on the earlier auxin minimum. Normal AM initiation requires cytokinin biosynthesis and signaling, as mutants of cytokinin biosynthetic genes (Müller et al., 2015), cytokinin receptor genes, and downstream B-type *ARABIDOPSIS RESPONSE REGULATOR* (*B-ARR*) TF genes (Wang et al., 2014b) compromise AM initiation. Also, STM can activate *IPT* expression to promote cytokinin biosynthesis (Jasinski et al., 2005; Yanai et al., 2005).

The existence of a linear regulatory pathway can be speculated, where the auxin minimum leads to *STM* expression, which enhances cytokinin biosynthesis and signaling. As cytokinins can act upstream of *STM* (Rupp et al., 1999), cytokinin and *STM* could establish a positive feedback loop during AM initiation. Furthermore, AM initiation also involves more-complicated regulatory interactions between phytohormones and TFs. For instance, exogenous cytokinin can restore AM initiation in *rax* mutants (Wang et al., 2014b). Also, ARR1, a TF downstream of cytokinin signaling, binds to the *LAS* promoter to promote its expression (Tian et al., 2014).

In addition to auxin and cytokinins, the leaf axil GRN also identified additional phytohormone signals that affect AM initiation (Tian et al., 2014). One candidate of particular interest is brassinosteroids (BRs). Recent findings have shown that BRs regulate organ boundary-specific gene expression and contribute to boundary formation (Bell et al., 2012; Gendron et al., 2012). Within the boundary zone, *LOB* can directly promote the expression of *PHYB ACTIVATION TAGGED SUPPRESSOR1* (*BAS1*), which encodes a BR-inactivating enzyme (Bell et al., 2012). Consistent with this, ectopic expression of *LOB* leads to a reduced BR response. Also, *LOB* dysfunction results in organ fusion, which can be suppressed by *BAS1* expression under the *LOB* promoter, indicating that BR hyper-accumulation contributes to boundary establishment in *lob* mutants (Bell et al., 2012). One hypothesis proposes that a local burst of BR signaling leads to activation of *LOB* expression, which in turn inactivates BR signaling (Arnaud and Laufs, 2013).

Of particular interest is the observation that the BR-activated gene *BRASSINAZOLE-RESISTANT1* (*BZR1*) represses *CUC3* via BZR1 directly binding to the *CUC3* promoter (Gendron et al., 2012). *CUC2* and *CUC3* positively regulate *LOF1* expression in the boundary zone (Gendron et al., 2012). Thus, *BZR1* indirectly causes the reduced expression of *LOF1*, possibly by repression of *CUC3*. Hence, the BR content decreases due to the *LOB*-dependent activation of *BAS1*, which results in a decrease of *BZR1* and an increase of *CUC* and *LOF1* levels in the boundary region (Bell et al., 2012; Gendron et al., 2012). Whether BR and its signaling affect AM initiation remains to be tested.

## Conclusion and Perspectives

Recent efforts to understand AM initiation have showed that various TFs and phytohormones collaboratively orchestrate initiation of AMs. However, many open questions still require further investigation. Like cells of the stomatal lineage, AM progenitor cells may represent a meristematic cell lineage. It will be important to understand the major shifts accompanying each fate transition from the designation of meristematic cells, to the maintenance of meristematic cell fate, and finally the activation of these cells to form new buds. For example, future work will address whether transcriptional regulation works in coordination with epigenetic mechanisms to affect cell fate determination during AM initiation. We also need to identify more regulators of AM initiation to obtain a comprehensive picture of this regulatory pathway.

## Author Contributions

ML and YJ wrote the manuscript.

## Conflict of Interest Statement

The authors declare that the research was conducted in the absence of any commercial or financial relationships that could be construed as a potential conflict of interest.
